# Autoantibodies Against Collapsin Response Mediator Proteins Associated With Encephalopathy/Myelopathy: A Single‐Center Retrospective Study

**DOI:** 10.1111/cns.70423

**Published:** 2025-06-29

**Authors:** Dongmei Wang, Qiqi Wang, Sanming Jie, Guanghui Liu, Xiaozhen Huang, Yue Pan, Kaibiao Xu, Chujun Chen, Yihua Huang, Yawei Jiang, Zirui Chen, Weiling Deng, Fenfen He, Chaowei Dai, Suyue Pan, Yongming Wu, Yafang Hu

**Affiliations:** ^1^ Department of Neurology, Nanfang Hospital Southern Medical University Guangzhou China; ^2^ Deptartment of Neurology The First Affiliated Hospital of Gannan Medical University Ganzhou China

**Keywords:** autoantibodies, biomarker, Collapsin response mediator proteins, encephalitis, encephalopathy

## Abstract

**Background:**

Collapsin response mediator protein (CRMP) consists of five subtypes (CRMP1–5), which share high homology and are expressed in the nervous system. Anti‐CRMP2 and anti‐CRMP5 antibodies (Abs) have been reported in autoimmune encephalitis (AE). This study retrospectively evaluated the diagnostic value of CRMP auto‐Abs in patients with suspected immune‐mediated encephalopathy/myelopathy.

**Methods:**

Patients with encephalopathy/myelopathy attributed to autoimmune or infectious causes, as well as those with encephalopathy of unclear etiology, were recruited from our department between January 2017 and November 2019. Clinical data, as well as serum and/or cerebrospinal fluid (CSF) samples, were collected. Measurement of Abs against CRMPs in patient samples was performed using a cell‐based assay (CBA) with HEK293 cells expressing CRMP proteins and confirmed by a tissue‐based assay (TBA) with mouse brain sections.

**Results:**

A total of 400 patients and 77 healthy controls were recruited. The male‐to‐female ratio of the patients was 0.88, and the average age was 39.22 ± 16.59 years. CBA testing was performed with 200 paired CSF and serum samples, along with 99 CSF samples and 101 serum samples. Of the patients, 22 (5.5%) presented with anti‐CRMPs Abs. Anti‐CRMP1 was the most commonly detected Ab (17/22, 77.3%), either alone or in combination with CRMP2 and CRMP3. Titers of anti‐CRMPs Abs ranged from 1: 3.2 to 1:10 in CSF samples and 1:32 to 1:320 in serum samples. Patients with anti‐CRMPs Abs experienced more headaches and had higher levels of chloride in CSF compared to those without anti‐CRMPs Abs. Fourteen of the 22 patients with anti‐CRMPs Abs were diagnosed with encephalitis, exhibiting a higher frequency of fever and headache, CSF pleocytosis, and more frequent treatment with immunotherapy, steroids, antibiotics, and antiviral therapy compared to non‐inflammatory encephalopathy patients.

**Conclusion:**

Anti‐CRMPs Abs may indicate immune‐mediated neuronal damage in encephalopathy, including encephalitis, and may serve as potential biomarkers for neuronal injury.

## Graphical abstract

This is a study to assess autoantibodies against CRMPs in a cohort of 400 patients with encephalopathy/myelopathy by CBA and TBA tests. Anti‐CRMPs Abs may indicate immune‐mediated neuronal damage in encephalopathy/myelopathy.

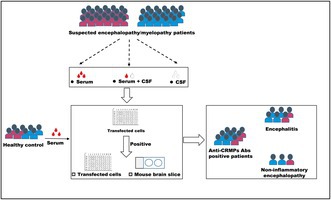

## 1 Introduction

The collapsin response mediator protein (CRMP) family consists of five intracellular subtypes (CRMP1–5), which can form homo‐ or hetero‐tetramers. The protein sequences of CRMP1–4 share approximately 75% homology, while CRMP5 exhibits about 50% homology with the other CRMPs [1]. CRMPs are predominantly expressed in the nervous system during development and remain significantly elevated in postnatal neurons, as well as in various types of cancer cells [1]. CRMPs play critical roles during embryonic development and in the postnatal refinement of the nervous system through posttranslational modifications, such as phosphorylation, and interactions with molecules such as microtubules, Sema3A, and reelin. These functions include cell migration, growth cone guidance, and axonal outgrowth [2, 3, 4, 5, 6]. Dysfunction of CRMPs due to pathological variants in *CRMP* genes has been linked to neurodevelopmental diseases. For example, monoallelic variants in the *CRMP1* gene cause a neurodevelopmental disorder characterized by muscular hypotonia, intellectual disability, and/or autism spectrum disorder [7]. Heterozygous variants in *CRMP5* lead to Ritscher–Schinzel syndrome 4 (MIM#619435), which is associated with craniofacial features, cerebral and cardiovascular malformations, and cognitive dysfunction [8]. Variants in *CRMP4* have been found in patients with amyotrophic lateral sclerosis [9].

Autoantibodies (auto‐Abs) against CRMPs have been studied in a range of autoimmune neurological disorders. Anti‐CV2/CRMP5‐IgG auto‐Ab is a well‐established diagnostic marker for paraneoplastic syndromes, commonly associated with small cell lung cancer. This syndrome is characterized by a diverse range of symptoms, including chorea, cerebellar ataxia, optic neuritis, peripheral and autonomic neuropathy, neuromuscular junction disorders, and cognitive dysfunction [10]. We had previously identified anti‐CRMP2‐IgG4 Abs in two patients who presented with acute cerebellar encephalitis or encephalomyelitis and responded well to immunotherapy [11]. Heat shock protein 60 and CRMP2 are autoantigens in autoimmune retinopathy and cancer‐associated retinopathy [12]. Antibodies against CRMP3–4 were detected in a case of subacute limbic encephalitis (LE) associated with thymoma [13]. Additionally, the presence of maternal auto‐Abs against CRMP1, CRMP2, lactate dehydrogenase A and B (LDH‐A, LDH‐B), guanine deaminase (GDA), stress‐induced phosphoprotein 1 (STIP1), and Y‐box binding protein 1 (YBX1), either individually or in combination, has been linked to maternal autoantibody‐related autism spectrum disorder (MAR ASD) [14, 15, 16]. Here, we conducted a single‐center retrospective study in patients with suspected immune‐mediated encephalopathy/myelopathy to assess the clinical diagnostic value of auto‐Abs against CRMPs.

## 2 Methods

### 2.1 Selection of Participants

**FIGURE 1:**
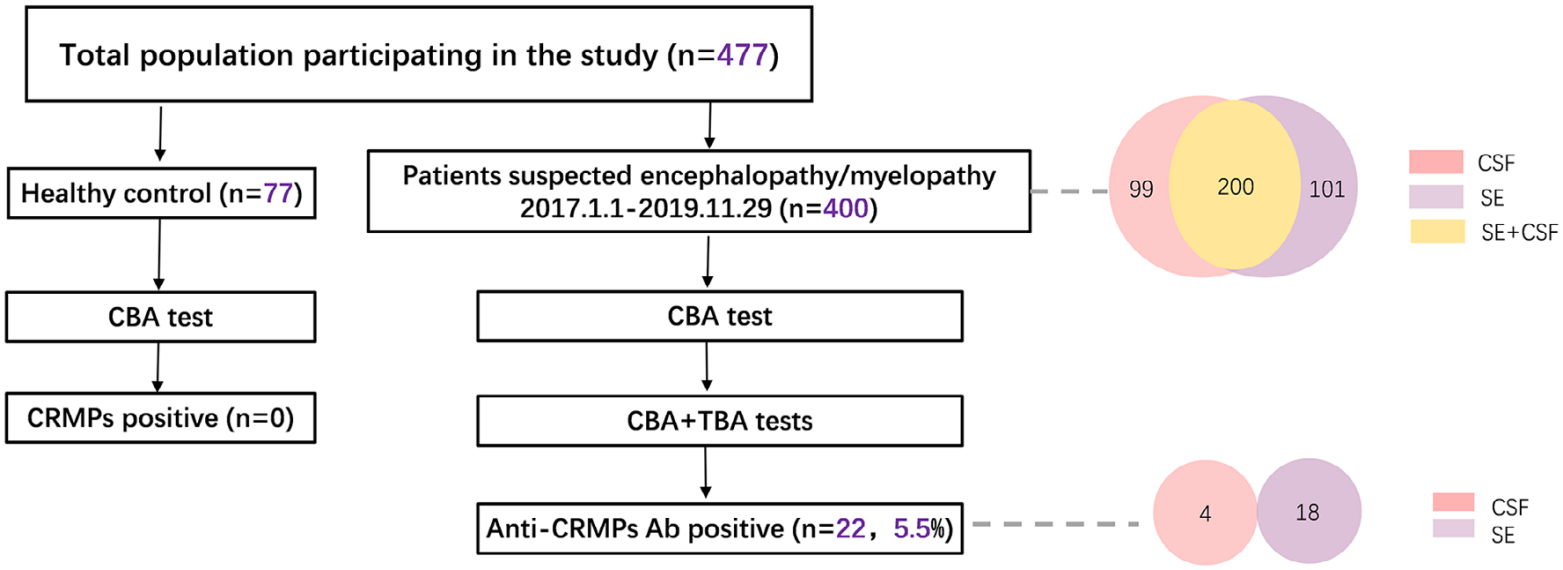
The inclusion flowchart of the patients. CBA, cell‐based assay; CRMPs, Collapsin response mediator proteins; CSF, cerebrospinal fluid; SE, Serum.

The inclusion criteria for patients are illustrated in Figure 1: (1) patients admitted to the Department of Neurology or the Neurocritical Unit (NCU) of Nanfang Hospital, Southern Medical University, between January 2017 and November 2019; (2) patients with suspected immune‐mediated encephalopathy (altered consciousness persisting for more than 24 h) [17] or myelopathy. This included cases of encephalopathy suspected to be autoimmune, infectious, or metabolic as well as encephalopathy with unclear etiology; (3) patients with remaining serum and/or cerebrospinal fluid (CSF) samples available for immunoreaction analysis using tissue‐based assay (TBA) and cell‐based assay (CBA). All samples were the first obtained from patients after hospitalization, prior to initiating treatment at our hospital. Some patients, however, may have received treatment at other hospitals before being transferred here. The exclusion criteria were: (1) loss to follow‐up; (2) incomplete data; (3) encephalopathy resulting from toxic, traumatic, anoxic/hypoxic, genetic, or endocrine causes.

This study was approved by the Ethical Review Committee of Nanfang Hospital (NFEC‐2021‐001; NFEC‐2022‐180). All patients or their proxies provided informed consent for the use of their remaining samples in the study.

### 2.2 Study Design and Setting

In this retrospective study, participants who met the inclusion criteria were identified through the electronic medical record system. The sociodemographic data, clinical features, treatments, cranial magnetic resonance imaging (MRI) results, and laboratory findings were collected. The duration of NCU stay and mechanical ventilation (MV) were also recorded. The modified Rankin Scale (mRS) was assessed at discharge and at 6 months during follow‐up. All patients were followed up by telephone or at the outpatient clinic.

### 2.3 TBA/Immunohistochemistry and CBA

TBA kits were prepared as follows: Anesthetized C57BL/6 mice were perfused with phosphate‐buffered saline (PBS), and the brains were carefully extracted. The brains were then embedded in optimal cutting temperature (OCT) compound (Sakura, Torrance, USA) and immediately frozen in liquid nitrogen. Brain sections with a thickness of 5 μm were cut using a Leica RM2245 microtome (Leica Microsystems, Germany). The sections were fixed with cold acetone for 20 min, dried, and stored at −20°C until use. Brain section slices were blocked with 1% BSA in PBST for 1 h at room temperature (RT), then incubated with 100 μL of serum samples (1:10 diluted with PBS), CSF samples (undiluted), or anti‐rabbit polyclonal CRMP Abs (1:200 dilution in PBS) at 37°C for 1 h. Anti‐CRMP Abs were provided by Proteintech (anti‐CRMP1, 2, 4, 5 Abs, Wuhan Sanying, Wuhan, China) or Affinity Biosciences (Anti‐CRMP3, Cincinnati, OH). The PBS‐washed slices were incubated with 100 μL of Alexa Fluor 488‐labeled goat anti‐human IgG or goat anti‐rabbit IgG‐H&L (Thermo Fisher, MA, 1:1000 dilution) at 37°C for 1 h, followed by three PBS washes. Fluorescence images were captured using an IX73 inverted microscope (Olympus, Japan). A TBA positive test was defined as the presence of green fluorescence in neuronal structures, such as the surface, cytosol, or nucleus, under 10x or 40x objective magnification.

The construction of expression plasmids (pcDNA3.1‐*CRMP* *1‐5*) and CBA tests were previously described [11]. Briefly, HEK293T cells were transfected with either the pcDNA3.1 vector or pcDNA3.1‐*CRMP1‐5* plasmids for 48 h. The transfected cells were fixed with ice‐cold acetone for 10 min, washed with PBST, blocked with 10% goat serum at RT for 1 h, and then incubated with the patient's serum (1:10 dilution in PBS, or 1:10, 1:32, 1:100, 1:320 dilution in PBS for titer calculation) or CSF samples (undiluted or 1:3.2, 1:10, 1:32 dilution in PBS for titer calculation) at 37°C for 1 h. After three PBS washes, fixed cells were incubated with DyLight 550‐labeled goat anti‐human IgG (1:200 dilution, Abcam) at 37°C for 30 min, followed by three PBS washes. Fluorescence images were viewed and captured under 20x/40x microscope using an IX73 inverted microscope (Olympus). A positive CBA test result was defined by the presence of at least 3–5 morphologically intact cells exhibiting distinct red fluorescence in every microscopic field observed under 20x objective magnification, and multiple fields of view were examined. The maximal dilution factor retaining positive reactivity was documented as the assay endpoint titer.

### 2.4 Statistical Analysis

All data analyses were performed using SPSS Statistics (version 25.0), with statistical significance set at *p* < 0.05. Categorical variables were expressed as numbers (%) and compared using either a two‐sided chi‐square test or Fisher's exact test. Continuous variables were expressed as mean ± standard deviation (SD) or median (interquartile range [IQR]) and were analyzed using an independent t‐test or Mann–Whitney U test.

## 3 Results

### 3.1 Demographical Information and Clinical Diagnosis of the Patients With Encephalopathy/Myelopathy

We recruited 400 patients with encephalopathy suspected to be autoimmune or infectious, as well as those with encephalopathy of unclear etiology. The male‐to‐female ratio was 0.88, and the average age was 39.22 ± 16.59 years. As shown in Figure 1, 200 patients had both serum and CSF samples, 99 patients had only CSF samples, and 101 patients and 77 healthy controls had serum samples. All samples were analyzed with CBA of HEK293 cells expressing human CRMP1‐5. Anti‐CRMP1‐5 Abs were detected in the serum samples of 18 patients and CSF samples of 4 patients (22/400, 5.5%), but not in paired samples. No anti‐CRMPs Abs were found in the healthy controls. Of the 22 patients, none were diagnosed with cancer.

**FIGURE 2:**
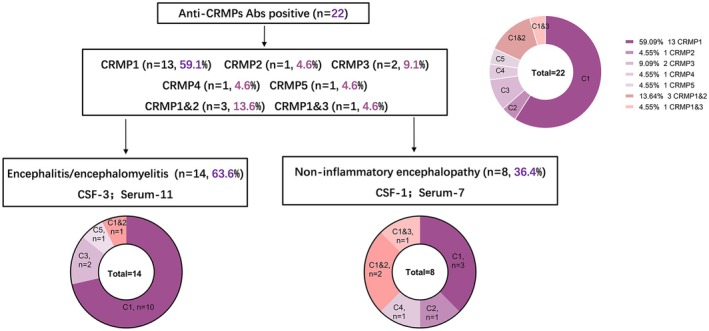
CRMPs antibody distribution of the patients. Of the 22 patients, anti‐CRMP1 antibodies were the most detected, followed by anti‐CRMP2 and anti‐CRMP3 antibodies. The distribution of anti‐CRMPs antibodies across the two diagnostic groups showed no significant difference. CRMPs, Collapsin response mediator proteins; CSF, cerebrospinal fluid.

Detailed information on the 22 patients with anti‐CRMPs antibodies is presented in Table 1. Based on clinical features and laboratory results, 14 patients (63.6%) were clinically diagnosed with encephalitis/encephalomyelitis, while 8 patients (36.4%) were diagnosed with non‐inflammatory encephalopathy/myelopathy (Figure 2). The CRMP subtypes involved in each case are also indicated.

**TABLE 1:** The detailed clinical data of 22 patients with positive anti‐CRMPs antibodies.

Patients	Gender	Age (years)	Diagnosis	Clinical symptoms	Sample type	Titer(subtype)	CSF			Immunotherapy	Other therapy	mRS
							WBC (cells/ul)	Glu (mmol/L)	Pro (g/L)			
P1	M	36	EB viral encephalomyelitis	Fever, dyssuresia, paralysis	SE	1:32 (C3)	10	2.5	0.95	Steroids, IVIG	Anti‐viral, antibiotics	5
P2	M	46	EB viral encephalitis	Fever, headache, blurred vision, somnolence	SE	1:32 (C1)	3	5.16	1.12	Steroids, IVIG	Anti‐viral, antibiotics	1
P3	M	17	Intracranial infection	Fever, seizures	SE	1:32 (C1)	0	3.31	0.28	None	Cefepime, valproate	0
P4	M	38	Brucella infection	Psychiatric disturbances	CSF	1:10 (C1)	1400	1.18	3.12	None	Antibiotics	2
P5	M	42	Cryptococcus encephalitis	Fever, headache	SE	1:100 (C1)	4	3.96	0.6	Steroids	Anti‐viral, antibiotics	1
P6	F	31	Meningitis with suspected bacterial infection	Headache, fever, nausea, vomiting	SE	1:100 (C1)	2449	1.53	0.97	None	Antibiotics	0
P7	M	44	Meningitis with suspected bacterial infection	Headache, fever	CSF	1:3.2 (C1)	92	2.95	0.76	None	Anti‐viral, ceftriaxone	1
P8	M	30	Meningoencephalitis, thyroid crisis	Psychiatric disturbances, cognitive dysfunction	SE	1:100 (C1)	99	2.25	0.7	Steroids, IVIG	Propylthiouracil, Anti‐viral, antibiotics	1
P9	F	36	Anti‐NMDAR encephalitis	Headache, fever, visual disturbance, psychiatric disturbances	SE	1:32 (C1)	0	5.41	0.21	IVIG, MP pulse, PE	None	4
P10	F	26	Anti‐NMDAR encephalitis	Psychiatric disturbance, cognitive dysfunction	SE	1:32 (C1)	27	3.76	0.2	Steroids, IVIG, PE	Anti‐viral, antibiotics, anti‐epileptic	4
P11	M	53	Autoimmune encephalitis (anti‐MOG antibody)	Headache	SE	1:32 (C1)	0	2.87	0.67	Steroids, IVIG	Anti‐viral, anti‐epileptic	1
P12	M	54	Autoimmune encephalitis (anti‐GFAP antibody)	Autonomic dysfunction (urinary disorders)	SE	1:100 (C1) 1:32 (C2)	2	2.53	0.36	Steroids	Anti‐viral, anti‐tuberculosis therapy	1
P13	F	24	Seizure with pregnancy	Seizure	CSF	1:3.2 (C5)	0	3.15	0.23	None	Topiramate and levetiracetam	1
P14	M	19	NORSE	Disturbance of consciousness, seizures	SE	1:32 (C3)	4	4.56	0.25	PE, steroids	Anti‐viral, antibiotics	4
P15	F	25	Multiple sclerosis	Dizziness, paralysis, blurred vision	SE	1:100 (C1)	7	3.31	0.31	Steroids	None	3
P16	F	44	Demyelinating encephalopathy	Dizziness, headache, paralysis	CSF	1:3.2 (C1)	0	4.04	0.52	None	Vit‐B	1
P17	M	27	Spinal cord lesion (demyelination)	Dizziness, visual disturbance	SE	1:32 (C1)	0	4.32	0.2	None	Vit‐B	1
P18	M	16	Seizure	Seizure	SE	1:32 (C2)	0	4.03	0.26	None	Anti‐viral, anti‐epileptic	1
P19	F	68	Parkinson's disease	Memory impairment, cognitive impairment	SE	1:32 (C1) 1:320 (C3)	NA	NA	NA	None	Regulating lipid, lowering blood sugar	1
P20	M	50	Wernicke encephalopathy	Nausea, dizziness, changed mental status	SE	1:32 (C1) 1:32 (C2)	0	3.13	0.26	None	Anti‐viral, anti‐epileptic, Vit‐B	1
P21	M	57	Subacute combined degeneration	Cough, expectoration, paralysis	SE	1:320 (C1) 1:100 (C2)	2	3.34	0.3	None	Vit‐B	1
P22	M	67	Encephalopathy	Fever, headache, changed mental status	SE	1:320 (C4)	0	2.86	0.9	None	Analgesia, levetiracetam	5

Abbreviations: CRMPs, collapsin response mediator proteins; CSF, cerebrospinal fluid; CT, computed tomography; EB, Epstein–Barr; F, female; GFAP, glial fibrillary acidic protein; Glu, glucose; IVIG, intravenous immunoglobulin; M, male; MOG, myelin oligodendrocyte glycoprotein; MRI, magnetic resonance imaging; mRS, modified Rankin Scale; NA, not available; PE, plasma exchange; Pro, protein; Vit‐B, B vitamins; WBC, white blood cell.

### 3.2 Titers and Distribution of CRMPs Auto‐Abs

The titers of anti‐CRMPs auto‐Abs in serum ranged from 1:32 to 1:320, and in CSF from 1:3.2 to 1:10 (Table 1). Among the CRMPs Abs, CRMP1 Abs were the most frequently detected, found in 60% (13/22) of patients, followed by co‐existence of CRMP1 and CRMP2 Abs (3/22, 13.64%), and CRMP3 Abs (2/22, 9.1%) (Figure 2). Among the 14 patients diagnosed with encephalitis/encephalomyelitis, 10 had CRMP1 Abs, 2 had anti‐CRMP3, 1 had anti‐CRMP5, and 1 had co‐existence of anti‐CRMP1 and anti‐CRMP2 Abs. Among the 8 patients with non‐inflammatory encephalopathy/myelopathy, 3 had anti‐CRMP1 Abs, 1 had anti‐CRMP2, 1 had anti‐CRMP4, 2 had co‐existence of anti‐CRMP1 and CRMP2 Abs, and 1 had co‐existence of anti‐CRMP1 and anti‐CRMP3 Abs. Of the 18 patients with positive serum CRMPs Abs, no significant correlation was observed between titers and mRS scores (*p* = 0.599).

**FIGURE 3:**
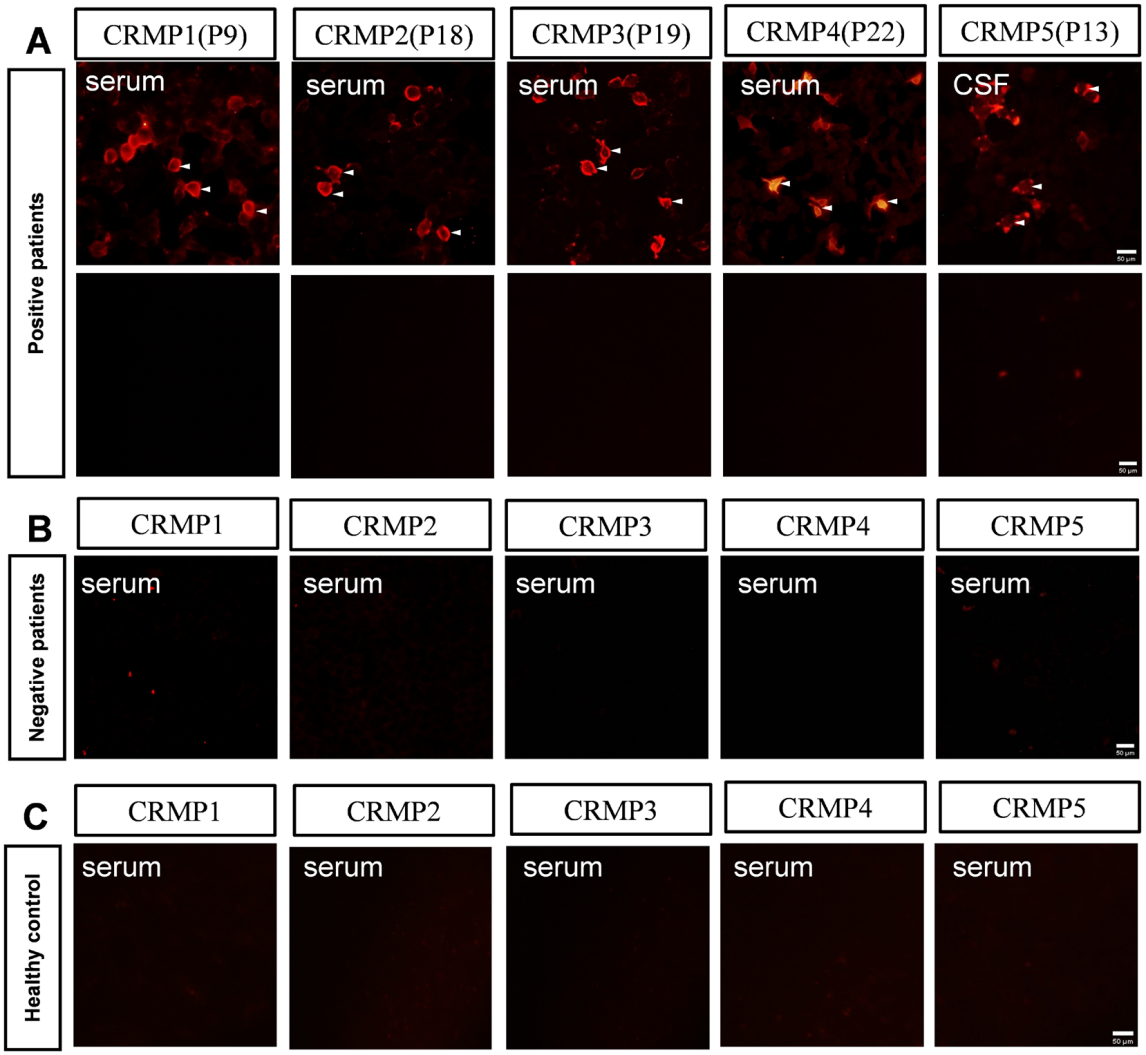
The CBA test of selected patients. (A) The CBA tests of serum or CSF from selected patients revealed anti‐CRMPs antibodies detected in the cytoplasm of HEK293 cells expressing different CRMPs, shown as red fluorescence staining (indicated by white arrows), upper panel. The same sample showed no red fluorescence staining in cells expressing the vector control, lower panel. (B and C) Serum samples from a selected patient without CRMPs Abs (B) or healthy controls (C) did not show red fluorescence staining in the cells expressing different CRMPs. CBA, cell based assay; CSF, cerebrospinal fluid; CRMPs, Collapsin response mediator proteins.

**FIGURE 4:**
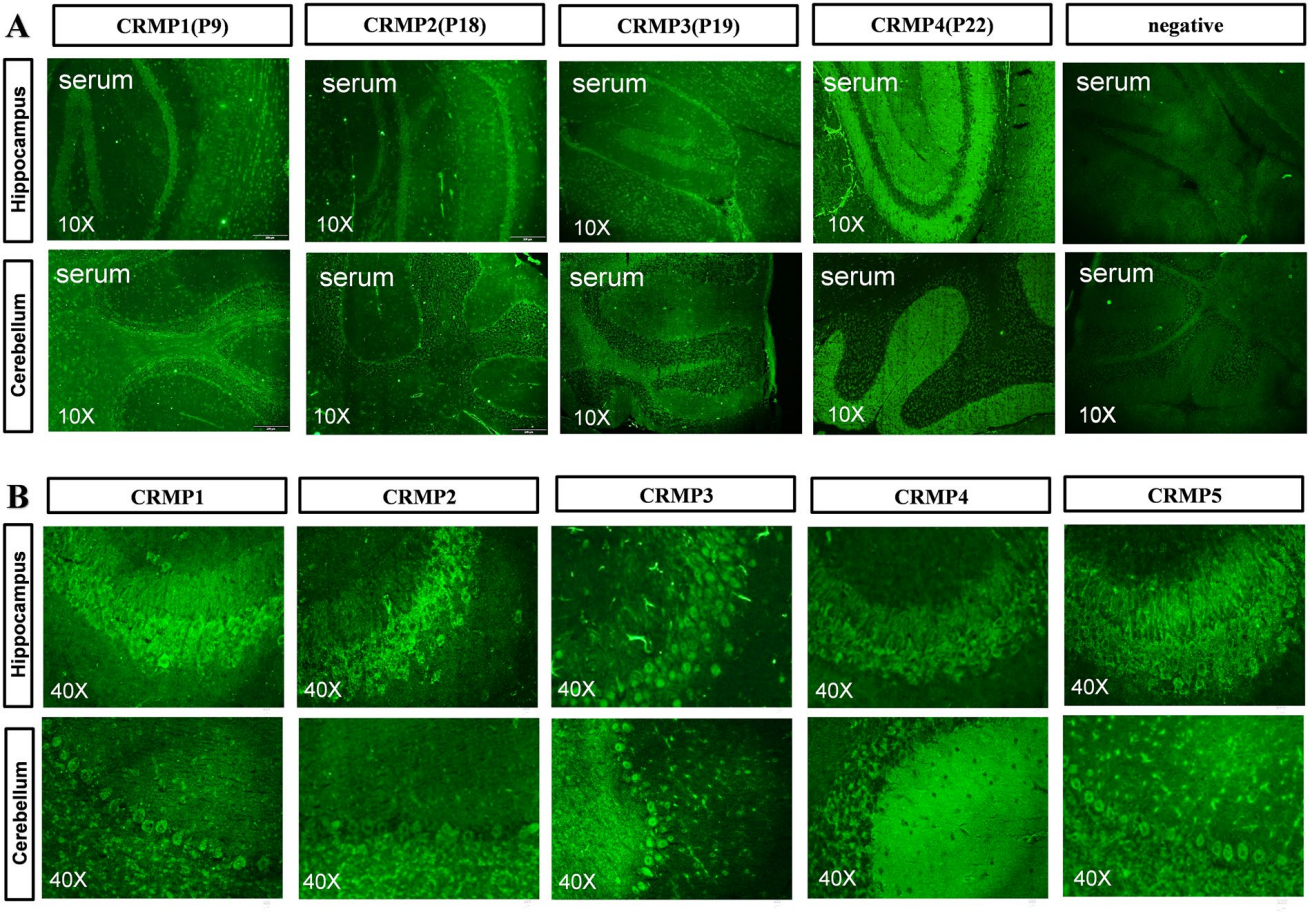
TBA/Immunohistochemistry test of selected patients. (A) Immunostaining from serum samples of selected patients was evident in the cytosolic compartments of neurons from the cortex, hippocampus, and cerebellum in TBA sections, as indicated by green fluorescence staining. This was not observed in the sample from patient 22, who had anti‐CRMP4 Abs, which showed neurofilament‐like staining. (B) TBA staining of commercial antibodies against five subtypes of CRMPs in the hippocampus and cerebellum of mice showed a similar pattern to the samples from patients with corresponding anti‐CRMPs Abs. CRMPs, Collapsin response mediator proteins; TBA, tissue‐based assay.

The CBA results are shown in Figure 3. Red fluorescence staining in the cytoplasm, indicated by arrows, represents positive auto‐Abs against overexpressed CRMP proteins (Figure 3A upper panel), while no signal was detected in vector control cells with the same sample (Figure 3A lower panel). TBA confirmed intracellular staining of CRMP1, 2, 3, and 5 Abs, as well as both intracellular and cell surface staining of CRMP4 Abs in neurons (Figure 4A). The staining patterns were consistent with those observed using corresponding commercial Abs (Figure 4B).

### 3.3 The Comparison of Clinical Features Between Patients With or Without Anti‐CRMPs Abs

The diagnoses of the 14 patients with encephalitis/encephalomyelitis and 8 patients with non‐inflammatory encephalopathy/myelopathy were based on clinical features, MRI findings, and CSF results, fulfilling the criteria for encephalitis or encephalopathy, respectively [17, 18, 19, 20, 21]. Since the patients' ages did not follow a normal distribution, we compared data between the 22 patients with anti‐CRMPs Abs and 44 age‐matched patients without anti‐CRMPs Abs (Table S1). As shown in Table 2, there were no differences between the two groups in terms of gender or age. However, patients with anti‐CRMPs Abs had a significantly higher percentage of headache (*p* = 0.034) and a higher chloride level (*p* = 0.032). No other significant differences were observed in prodromal symptoms, clinical symptoms, laboratory tests, treatment, or outcomes.

**TABLE 2:** The comparisons of clinical features between patients with or without anti‐CRMPs Abs.

Variables	With anti‐CRMPs Abs (*n* = 22)	Without anti‐CRMPs Abs (*n* = 44)	*p*‐value
*Sociodemographic information*			
Female, *n* (%)	8 (36.4%)	16 (36.4%)	1.000
Age (years), median [IQR]	39 [26–51]	38 [26–52]	0.954
*Prodromal symptoms*			
Fever, *n* (%)	12 (54.6%)	24 (54.6%)	1.000
Headache, *n* (%)	13 (59.1%)	14 (31.8%)	0.034^a^
Nausea and vomiting, *n* (%)	3 (13.6%)	9 (20.5%)	0.735
*Clinical symptoms*			
Disturbance of consciousness, *n* (%)	9 (40.9%`)	15 (34.1%)	0.587
Cognitive dysfunction, *n* (%)	1 (4.6%)	4 (9.1%)	0.869
Motor disorder, *n* (%)	5 (22.7%)	15 (34.1%)	0.344
Psychiatric symptoms, *n* (%)	5 (22.7%)	11 (25%)	0.839
Seizure, *n* (%)	3 (13.6%)	12 (27.3%)	0.213
Speech disturbance, *n* (%)	4 (18.2%)	12 (27.3%)	0.417
Involuntary movement, *n* (%)	8 (36.4%)	17 (38.4%)	0.858
Autonomic nervous dysfunction, *n* (%)	4 (18.2%)	4 (9.1%)	0.505
Paresthesia, *n* (%)	1 (4.6%)	6 (13.6%)	0.480
Paralysis, *n* (%)	1 (4.6%)	2 (4.6%)	0.531
*Laboratory results*			
CSF white blood cell (cells/μL), median [IQR]	195 [0–19]	42 [0–60]	0.839
CSF glucose (mmol/L), median [IQR]	3.34 [2.70–4.04]	3.19 [2.75–4.00]	0.593
CSF protein (g/L), median [IQR]	0.63 [0.26–0.83]	0.81 [0.26–1.02]	0.593
CSF chloride (mmol/L), median [IQR]	125.4 [124.3–128.8]	121.2 [116.3–128.3]	0.032^a^
*Treatment*			
Immunotherapy, *n* (%)	10 (45.6%)	27 (61.4%)	0.220
Immunosuppressant, *n* (%)	2 (9.1%)	2 (4.6%)	0.855
Plasmapheresis, *n* (%)	2 (9.1%)	0 (0%)	0.108
Steroids, *n* (%)	10 (45.5%)	25 (56.8%)	0.383
IVIG, *n* (%)	5 (22.7%)	7 (15.9%)	0.735
Antibiotics, *n* (%)	11 (50%)	19 (43.2%)	0.600
Antiviral therapy, *n* (%)	8 (36.4%)	14 (31.8%)	0.712
*Outcomes*			
Ventilation time (days), median [IQR]	1 [0–0]	1 [0–0]	0.939
LOS (days) median [IQR]	21 [9–27]	14 [8–19]	0.107
Length of NCU stay (days), median [IQR]	3 [0–0]	2 [0–0]	0.851
Good outcome (mRS ≤ 2), discharge, *n* (%)	16 (72.7%)	38 (86.4%)	0.310

Abbreviations: CRMPs, collapsin response mediator proteins; CSF, cerebrospinal fluid; IQR, interquartile range; IVIG, Intravenous immunoglobulin; LOS, length of hospital stay; mRS, modified Rankin Scale; NCU, neurocritical care unit.

^a^ Indicates a statistically significant difference.

Further comparison between encephalitis/encephalomyelitis and non‐inflammatory encephalopathy/myelopathy patients with anti‐CRMPs Abs is provided in Table 3. The two groups were similar in gender and age. Regarding prodromal symptoms, patients with encephalitis/encephalomyelitis had a significantly higher percentage of fever and headache (*p* = 0.006, *p* = 0.026, respectively). No significant differences were observed in clinical symptoms (Table 3). CSF laboratory tests showed significant pleocytosis in the encephalitis group, but no significant differences were found between the two groups in terms of glucose, protein, and chloride levels. The encephalitis/encephalomyelitis group received higher percentages of immunotherapy, antibiotics, and antiviral therapy (*p* = 0.031, *p* = 0.024, *p* = 0.018, respectively). No significant differences in outcomes were found between the two groups.

**TABLE 3:** The comparisons of clinical features between patients with encephalitis/encephalomyelitis and non‐inflammatory encephalopathy/myelopathy.

Variables	Encephalitis/encephalomyelitis (*n* = 14)	Non‐inflammatory (*n* = 8)	*p*‐value
*Sociodemographic information*			
Female, *n* (%)	5 (35.7%)	3 (37.5%)	1.000
Age (years), median [IQR]	36 [26–45]	47 [26–65]	0.277
*Prodromal symptoms*			
Fever, *n* (%)	11 (78.6%)	1 (12.5%)	0.006^a^
Headache, *n* (%)	11 (78.6%)	2 (25%)	0.026^a^
Nausea and vomiting, *n* (%)	2 (14.3%)	1 (12.5%)	1.000
*Clinical symptoms*			
Disturbance of consciousness, *n* (%)	6 (42.9%)	3 (37.5%)	1.000
Cognitive dysfunction, *n* (%)	0 (0%)	1 (12.5%)	0.364
Motor disorder, *n* (%)	3 (21.4%)	2 (25%)	1.000
Psychiatric symptoms, *n* (%)	5 (35.7%)	0 (0%)	0.115
Seizure, *n* (%)	2 (14.3%)	1 (12.5%)	1.000
Speech disturbance, *n* (%)	2 (14.3%)	2 (25%)	0.602
Involuntary movement, *n* (%)	6 (42.9%)	2 (25%)	0.649
Autonomic nervous dysfunction, *n* (%)	3 (21.4%)	1 (12.5%)	1.000
Paresthesia, *n* (%)	0 (0%)	1 (12.5%)	0.364
Paralysis, *n* (%)	0 (0%)	1 (12.5%)	0.364
*Laboratory results*			
CSF white blood cell (cells/μL), median [IQR]	4 [0–94]	0 [0–2]	0.039^a^
CSF glucose (mmol/L), median [IQR]	3.05 [2.44–4.11]	3.34 [3.13–4.04]	0.381
CSF protein (g/L), median [IQR]	0.64 [0.25–0.96]	0.3 [0.26–0.52]	0.279
CSF chloride (mmol/L), median [IQR]	126.1 [121.4–128.8]	127.2 [126.8–128.9]	0.220
*Treatment*			
Immunotherapy, *n* (%)	9 (64.3%)	1 (12.5%)	0.031^a^
Immunosuppressant, *n* (%)	2 (14.3%)	0 (0%)	0.515
Plasmapheresis, *n* (%)	2 (14.3%)	0 (0%)	0.515
Steroids, *n* (%)	9 (64.3%)	1 (12.5%)	0.031^a^
IVIG, *n* (%)	5 (51.0%)	0 (0%)	0.115
Antibiotics, *n* (%)	10 (71.4%)	1 (12.5%)	0.024^a^
Antiviral therapy, *n* (%)	8 (57.1%)	0 (0%)	0.018^a^
*Outcomes*			
Ventilation time (days), median [IQR]	0 [0–0]	0 [0–0]	0.274
LOS (days) median [IQR]	21 [14–42]	11 [9–14]	0.065
Length of NCU stay (days), median [IQR]	0 [0–10]	0 [0–0]	0.105
Good outcome (mRS ≤ 2), discharge, *n* (%)	10 (71.4%)	6 (75.0%)	1.000

Abbreviations: CSF, cerebrospinal fluid; IQR, interquartile range; IVIG, Intravenous immunoglobulin; LOS, length of hospital stay; mRS, modified Rankin Scale; NCU, neurocritical care unit.

^a^ Indicates a statistically significant difference.

Among the 14 patients with encephalitis/encephalomyelitis, eight were classified as having an infection. These included one patient diagnosed with cryptococcal encephalomyelitis, one with influenza infection, two with Epstein–Barr virus encephalitis, and four with purulent infections (one with *Streptococcus pneumoniae* , one with brucellosis, and two with an unknown pathogen). In the non‐infectious group, diagnoses included two cases of anti‐N‐methyl‐d‐aspartate (NMDA) receptor encephalitis, one case of myelin oligodendrocyte glycoprotein IgG‐associated disorder (MOGAD), one case of anti‐glial fibrillary acidic protein (GFAP)‐related astrocytopathy, one case of seizure of unknown origin, and one case of new‐onset refractory status epilepticus (NORSE). When comparing the infectious and non‐infectious groups, no significant differences were found in sociodemographic information, prodromal symptoms, or neurological symptoms. However, patients in the infectious group showed significantly higher protein levels (*p* = 0.007), relatively higher WBC counts in CSF (*p* = 0.050), and a higher percentage of antibiotic use (*p* = 0.015). Immunotherapy was initiated in most patients in both groups. No significant differences in outcomes were observed between the two groups (Table 4).

**TABLE 4:** The comparisons of clinical features between patients with infectious and non‐infectious encephalitis/encephalomyelitis.

Variables	Infectious (*n* = 8)	Non‐infectious (*n* = 6)	*p*‐value
*Sociodemographic information*			
Female, *n* (%)	2 (25%)	3 (50%)	0.580
Age (years), median [IQR]	37 [30–44]	31 [23–53]	0.982
*Prodromal symptoms*			
Fever, *n* (%)	8 (100%)	3 (50%)	0.055
Headache, *n* (%)	7 (87.5%)	4 (66.7%)	0.538
Nausea and vomiting, *n* (%)	2 (25%)	0 (0%)	0.473
*Clinical symptoms*			
Disturbance of consciousness, *n* (%)	3 (37.5%)	3 (50%)	1.000
Motor disorder, *n* (%)	1 (12.5%)	2 (33.3%)	0.538
Psychiatric symptoms, *n* (%)	2 (25%)	3 (50%)	0.580
Seizure, *n* (%)	1 (12.5%)	1 (16.7%)	1.000
Speech disturbance, *n* (%)	1 (12.5%)	1 (16.7%)	1.000
Involuntary movement, *n* (%)	2 (25%)	4 (66.7%)	0.277
Autonomic nervous dysfunction, *n* (%)	1 (12.5%)	2 (33.3%)	0.538
*Laboratory results*			
CSF white blood cell (cells/μL), median [IQR]	51 [3–1075]	1 [0–10]	0.050
CSF glucose (mmol), median [IQR]	2.73 [1.71–3.80]	3.46 [2.79–4.77]	0.206
CSF protein (g/L), median [IQR]	0.86 [0.63–1.08]	0.24 [0.21–0.44]	0.007^a^
CSF chloride^−^ (mmol/L), median [IQR]	124.9 [112.2–126.6]	127.3 [121.5–129.5]	0.303
*Treatment*			
Immunotherapy, *n* (%)	4 (50%)	5 (83.3%)	0.301
Plasmapheresis, *n* (%)	0 (0%)	2 (33.3%)	0.165
Steroids, *n* (%)	4 (50.0%)	5 (83.3%)	0.301
IVIG, *n* (%)	3 (37.5%)	2 (33.3%)	1.000
Antibiotics, *n* (%)	8 (100%)	2 (33.3%)	0.015^a^
Antiviral therapy, *n* (%)	5 (62.5%)	3 (50.0%)	1.000
*Outcomes*			
Ventilation time (days), median [IQR]	0 [0–0]	0 [0–4]	0.751
LOS (days) median [IQR]	18 [8–21]	31 [19–54]	0.093
Length of NCU stay (days), median [IQR]	0 [0–7]	0 [0–15]	0.628
Good outcome (mRS ≤ 2), discharge, *n* (%)	7 (87.5%)	3 (50%)	0.245

Abbreviations: CSF, cerebrospinal fluid; IQR, interquartile range; IVIG, Intravenous immunoglobulin; LOS, length of hospital stay; mRS, modified Rankin Scale; NCU, neurocritical care unit.

^a^ Indicates a statistically significant difference.

### 3.4 Imaging Findings of the Patients With CRMPs Auto‐Abs

**FIGURE 5:**
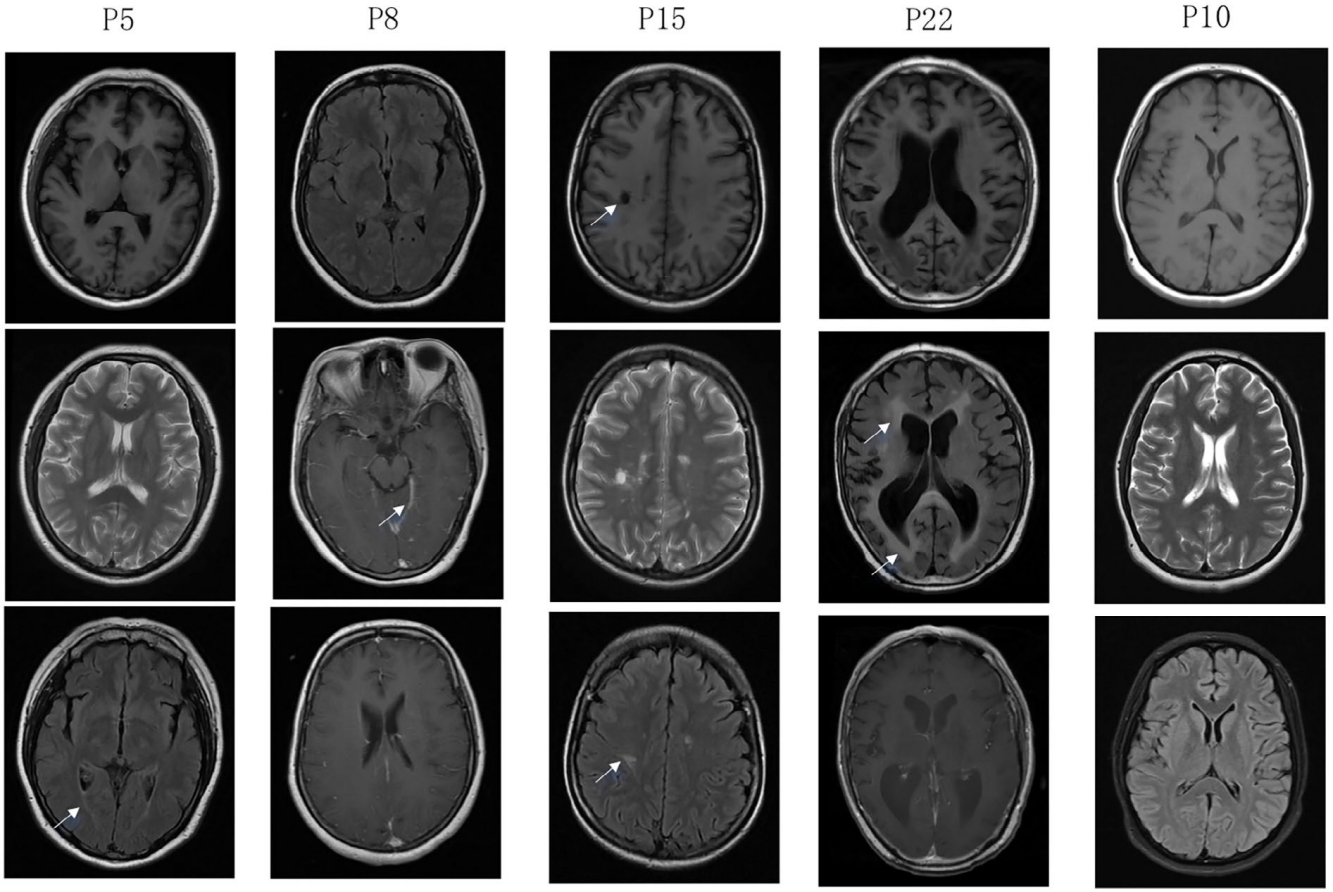
The image findings of the included patients. Cranial MRI of P5 revealed mild white matter degeneration (indicated by arrow). Cranial MRI of P8 suggested thickening of the bilateral cerebellar tentorium. In P15, multiple abnormal signals were observed in T1, T2, and FLAIR, suggesting demyelinating changes (indicated by arrows). Diffuse white matter changes were observed in the periventricular white matter (indicated by arrows) without enhancement in P22's cranial MRI. Cranial MRI of P10 was normal. FLAIR, fluid‐attenuated inversion recovery; MRI, magnetic resonance imaging.

In the encephalitis/encephalomyelitis group (P1‐14), the primary MRI change in the infectious group was meningeal enhancement. In contrast, the autoimmune encephalitis group exhibited scattered white matter degeneration or mild brain atrophy. Mild brain atrophy was also observed in the seizure and NORSE patients (Table S2 and Figure 5). In the non‐inflammatory encephalopathy/myelopathy group (P15‐22), the main MRI findings included demyelinating lesions in the cerebral or spinal cord. One patient diagnosed with encephalopathy of unknown origin showed diffuse leukoencephalopathy involving the bilateral frontal lobes, periventricular white matter, and corona radiata (Figure 5).

### 3.5 Information of Immunotherapy

As shown in Table 2, there was no significant difference in the administration of immunotherapy, including steroids, intravenous immunoglobulin, plasma exchange, or immunosuppressants, between patients with and without CRMPs Abs. Among the 22 patients with CRMPs Abs, 10 received immunotherapy. In the encephalitis/encephalomyelitis group, nine patients received immunotherapy, whereas only one patient in the non‐inflammatory encephalopathy/myelopathy group received it. The percentage of patients receiving immunotherapy was significantly higher in the encephalitis/encephalomyelitis group compared to the non‐inflammatory encephalopathy/myelopathy group.

## 4 Discussion

In this single‐center retrospective study, we tested anti‐CRMPs Abs in 400 patients with suspected encephalopathy/myelopathy, including encephalitis/encephalomyelitis. We found that 5.5% (22/400) of the patients tested positive for anti‐CRMPs Abs. The titers of anti‐CRMPs Abs ranged from 1:32 to 1:320 in serum and from 1:3.2 to 1:10 in CSF. Anti‐CRMP1 Abs were the most commonly detected, either alone (13/22, 59.1%) or coexisting with other anti‐CRMPs Abs (4/22, 18.2%). Patients with anti‐CRMPs Abs had more frequent headaches and higher chloride levels in CSF compared to patients without any anti‐CRMPs Abs. The association between chloride levels and anti‐CRMPs Abs remains unclear. Of the 22 patients with anti‐CRMPs Abs, 14 were clinically diagnosed with encephalitis/encephalomyelitis. These patients tended to present with fever and headache as prodromal symptoms, CSF pleocytosis, longer hospital stays, and a higher likelihood of receiving immunotherapy.

Previously, we demonstrated that anti‐CRMP2 Ab was associated with encephalitis/encephalomyelitis and that immunotherapy was beneficial for these patients [11]. Active immunization of mice with human CRMP2 produced anti‐CRMP2 Abs and resulted in unstable posture on a balance beam and anxiety‐like behaviors [22]. Additionally, anti‐CRMP2 Abs increased neural excitability, suggesting that these antibodies may dysregulate neuronal function. Several auto‐Abs targeting intercellular but synaptic‐localized proteins, such as GAD65, amphiphysin, and synapsin, have been implicated in pathogenesis [23, 24, 25, 26]. In the present study, four patients tested positive for anti‐CRMP2 Abs. Of these, one patient (P18) had only anti‐CRMP2 Abs, while three others (P12, P20, P21) had coexisting anti‐CRMP1 Abs. The clinical presentation of P18 included seizures, with no evidence of pleocytosis in the CSF, which differed from our previous patients who had definite evidence of encephalitis/encephalomyelitis. The other three patients with coexisting anti‐CRMP1 and anti‐CRMP2 Abs were diagnosed with autoimmune encephalitis (anti‐GFAP antibody), possible Wernicke encephalopathy, and subacute combined degeneration, respectively.

Anti‐CRMP1 Ab was the most frequently detected antibody in this study. Peptide microarray experiments have shown that the antigenic epitope of CRMP1 can be recognized and bound by plasma samples from the mothers of children with autism spectrum disorders [27]. Additionally, animal studies have confirmed that rats exposed to MAR‐ASD Abs exhibit alterations in behavior, brain structure, and neurometabolites [28]. In our study, the clinical features of patients with anti‐CRMP1 Abs varied, with no specific pattern observed. Anti‐CRMP5, also known as anti‐CV2 antibody, has been implicated in paraneoplastic syndromes [29]. Patients with anti‐CV2 antibodies often present with encephalitis, chorea, dystonia, and cerebellar ataxia [30, 31]. Furthermore, anti‐CRMP5 Abs have been reported as coexisting antibodies in other paraneoplastic neurological syndromes [32, 33]. In this study, one patient (P13) tested positive for anti‐CRMP5 Ab and presented with seizures. She was pregnant and treated with anti‐epileptic therapy, with gradual improvement of symptoms. However, no tumor was detected during follow‐up. It remains unclear whether anti‐CRMPs Abs are biomarkers of autoimmunity or simply indicators of neuronal damage. The exact role of anti‐CRMPs Abs warrants further investigation.

In this study, we identified four patients (P9‐P12) diagnosed with AE who had coexisting anti‐CRMPs Abs. Two of these patients were diagnosed with anti‐NMDAR encephalitis, one had anti‐MOG Ab, and the other had anti‐GFAP Ab. Previous studies have suggested the presence of coexisting Abs in AE patients [34, 35, 36, 37], which aligns with our findings [38]. However, no specific clinical pattern emerged among these patients. Most symptoms could be explained by known causes, except for two patients diagnosed with seizures of unknown origin and NORSE. Autoimmune‐mediated mechanisms and immunotherapy [39, 40] have been reported in studies of NORSE, where unknown antibodies have been detected, though their roles remain unclear [39, 41]. Based on our findings, we suggest that autoimmune‐mediated mechanisms cannot be excluded in these cases.

In this study, several patients were diagnosed with infections. Infection is a risk factor for AE, and secondary autoimmune damage might be triggered [42, 43]. Clinically, we treat severe intracranial infections, such as bacterial, viral, and cryptococcal infections, with steroids to control the inflammatory storm while targeting the pathogens simultaneously, and patients experience benefits from the therapy even during long‐term follow‐up [44, 45]. In the present study, we treated more than half of the infectious patients with steroids, which was beneficial to most patients.

This study has several limitations. First, this was a retrospective study using residual samples, which led to incomplete tests of both serum and CSF. Secondly, as a tertiary teaching hospital, many patients were transferred from other hospitals, introducing inevitable bias into the data. Thirdly, the sample size was relatively small, and the follow‐up time was short. Lastly, we did not perform an appropriate multiple comparison procedure to prevent the risk of false positive inferences. Therefore, the results should be interpreted with caution. More research is warranted to elucidate the mechanism of the antibodies.

## 5 Conclusions

CRMPs are widely distributed in the CNS, and anti‐CRMPs Abs have been detected in up to 5.5% of patients with suspected encephalopathy/myelopathy. Anti‐CRMPs Abs might be potential biomarkers for neuronal damage.

## Conflicts of Interest

The authors declare no conflicts of interest.

## Supporting information


Table S1.

Table S2.

## Data Availability

The data that support the findings of this study are available on request from the corresponding author. The data are not publicly available due to privacy or ethical restrictions.
