# Supplementary material for: Autoantibodies Against Collapsin Response Mediator Proteins Associated With Encephalopathy/Myelopathy: A Single‐Center Retrospective Study
Source: CNS Neurosci Ther. 2025 Jun 29;31(6):e70423. doi: 10.1111/cns.70423 (PMC12206659; doi:10.1111/cns.70423)
Supplement: Supplementary file 1 — Table S1. [file CNS-31-e70423-s001.docx]

**Supplementary Table 1 The detailed clinical data of 44 patients without anti-CRMPs antibodies.**

| Patients | Gender | Age (years) | Diagnosis | Clinical symptoms | CSF | | | | Immunotherapy | Other therapy | mRS |
| --- | --- | --- | --- | --- | --- | --- | --- | --- | --- | --- | --- |
|  |  |  |  |  | **WBC (cells/ul)** | **Glu (mmol/L)** | **Pro**  **(g/L)** | **Cl^-^（mmol/L）** |  |  |  |
| P23 | M | 40 | EB viral encephalomyelitis | Fever, nausea, vomiting, paresthesia | 120 | 2.77 | 1.21 | 110 | Steroids, IVIG | Anti-viral, antibiotics | 0 |
| P24 | M | 45 | Intracranial infection | Headache, fever, disturbance of consciousness, psychiatric symptoms, seizure, involuntary movement | 260 | 0.38 | 2.7 | 119.9 | None | Antibiotics | 5 |
| P25 | M | 38 | Intracranial infection | Fever, disturbance of consciousness, seizure | 32 | 2.86 | 0.55 | 126.7 | IVIG | Antibiotics | 0 |
| P26 | M | 26 | Viral encephalomyelitis | Fever, psychiatric symptoms, involuntary movement | 0 | 4.02 | 0.26 | 129.9 | IVIG | Antibiotics | 0 |
| P27 | M | 17 | Anti-NMDAR encephalitis | Fever, seizure, involuntary movement | 0 | 3.17 | 0.26 | 126.1 | Steroids | Anti-epileptic | 0 |
| P28 | M | 19 | Intracranial infection | Headache, fever, nausea, vomiting, disturbance of consciousness | 130 | 1.25 | 2.56 | 109 | Steroids | Antibiotics | 1 |
| P29 | M | 35 | Acute disseminated encephalomyelitis | Headache, fever, disturbance of consciousness, motor disorder, seizure, involuntary movement | 26 | 3.19 | 0.5 | 109.2 | Steroids | Anti-viral, antibiotics | 1 |
| P30 | M | 45 | Intracranial infection | Headache, nausea, vomiting， | 1 | 2.82 | 0.34 | 128.6 | None | Anti-viral | 1 |
| P31 | M | 47 | Cryptococcus meningoencephalitis | Headache, fever, disturbance of consciousness | 60 | 2.88 | 2.63 | 120.6 | Steroids | Anti-viral, antibiotics | 1 |
| P32 | M | 55 | Cryptococcus meningitis | Headache, nausea, vomiting， | 40 | 1.69 | 2.29 | 109.5 | None | Antibiotics | 0 |
| P33 | F | 25 | Intracranial infection | Fever, disturbance of consciousness, motor disorder, involuntary movement | 0 | 4.03 | 1.3 | 112.8 | Steroids | Antibiotics | 1 |
| P34 | F | 30 | Encephalitis | Motor disorder, psychiatric symptoms, speech disturbance | 2 | 2.7 | 0.27 | 120 | None | Anti-viral | 2 |
| P35 | M | 58 | Encephalitis | Fever, motor disorder, speech disturbance | 70 | 6 | 1.02 | 122.8 | Steroids | Anti-viral, antibiotics | 1 |
| P36 | M | 36 | Tuberculous meningitis | Headache, fever, nausea, vomiting, involuntary movement | 78 | 0.95 | 2.79 | 113.7 | Steroids | Anti-viral, anti-tuberculosis | 1 |
| P37 | F | 26 | Encephalitis | Headache, fever, nausea, vomiting， | 100 | 0.87 | 0.72 | 110.6 | Steroids | Anti-viral, antibiotics | 0 |
| P38 | F | 20 | Anti-NMDAR encephalitis | Psychiatric symptoms, speech disturbance | 0 | 3.6 | 0.25 | 131.2 | Steroids, IVIG | Anti-viral | 1 |
| P39 | F | 33 | Anti-NMDAR encephalitis | Fever, psychiatric symptoms, involuntary movement | 0 | 3.47 | 0.19 | 118.8 | Steroids | None | 0 |
| P40 | F | 34 | encephalitis | Headache, fever, disturbance of consciousness, motor disorder, seizure, involuntary movement, speech disturbance | 0 | 2.77 | 0.13 | 124.5 | Steroids | Antibiotics | 1 |
| P41 | F | 29 | Anti-NMDAR encephalitis | Seizure | 0 | 3.53 | 0.17 | 129.5 | Steroids, IVIG | Anti-viral, antibiotics | 0 |
| P42 | F | 40 | Meningitis | Headache, fever, nausea, vomiting | 1 | 1.41 | 1.68 | 105.8 | Steroids | Antibiotics | 2 |
| P43 | M | 60 | Anti-GFAP encephalitis | Fever, nausea, vomiting, hypopsia | 148 | 2.64 | 1 | 116.3 | Steroids, IVIG | None | 1 |
| P44 | M | 64 | Anti-NMDAR encephalitis | Fever, motor disorder, speech disturbance | 0 | 3.99 | 0.53 | 118.4 | Steroids | None | 0 |
| P45 | M | 51 | HSV-1 viral encephalitis | Headache, fever, disturbance of consciousness, speech disturbance | 64 | 2.93 | 0.99 | 131.5 | None | Antibiotics | 2 |
| P46 | M | 57 | Anti-NMDAR encephalitis | Disturbance of consciousness, psychiatric symptoms, seizure, involuntary movement | 2 | 3.98 | 0.47 | 124.2 | Steroids, IVIG, Azathioprine | None | 1 |
| P47 | F | 23 | Seizure | Disturbance of consciousness, seizure, involuntary movement | 0 | 2.99 | 0.2 | 117.2 | None | Anti-epileptic | 0 |
| P48 | F | 22 | Seizure | Cognitive dysfunction, seizure | 1 | 3.15 | 0.18 | 126.4 | None | Anti-epileptic | 0 |
| P49 | M | 17 | Seizure | Nausea, vomiting， | 0 | 3.32 | 0.22 | 129.9 | None | Anti-epileptic | 1 |
| P50 | M | 18 | Hemophagocytic lymphohistiocytosis | Headache, fever, disturbance of consciousness, seizure, involuntary movement, speech disturbance | 10 | 3.09 | 0.24 | 106.4 | Steroids, IVIG | Anti-viral, antibiotics, anti-epileptic | 5 |
| P51 | F | 27 | Demyelinating encephalopathy | Fever, motor disturbance, speech disturbance, involuntary movement | 20 | 2.9 | 0.67 | 119.2 | Steroids, IVIG | Anti-viral, antibiotics | 3 |
| P52 | F | 30 | Neuromyelitis spectrum | Motor disorder | NA | NA | NA | NA | Steroids | None | 1 |
| P53 | F | 49 | Demyelinating encephalopathy | Motor disorder, psychiatric symptoms, involuntary movement | 0 | 2.58 | 0.7 | 124.1 | Steroids | None | 3 |
| P54 | F | 46 | Multiple sclerosis | Paresthesia, paralysis | 0 | 3.4 | 0.24 | 128.3 | Steroids, IVIG | None | 0 |
| P55 | M | 27 | Demyelinating pseudotumor | Motor disorder, speech disturbance | 0 | 3.37 | 0.61 | 130.4 | Steroids, IVIG | None | 2 |
| P56 | M | 31 | Demyelinating encephalopathy | Fever, motor disorder | 4 | 4.46 | 0.61 | 122.4 | Steroids, IVIG | Antibiotics | 0 |
| P57 | M | 20 | Intracranial infection | Headache, fever, disturbance of consciousness, seizure, involuntary movement | 460 | 2.55 | 1.17 | 125.5 | None | Anti-viral, antibiotics | 0 |
| P58 | M | 17 | Seizure | Disturbance of consciousness, psychiatric symptoms, seizure, involuntary movement | NA | NA | NA | NA | None | Anti-viral, antibiotics | 1 |
| P59 | F | 71 | Metabolic encephalopathy | Fever, psychiatric symptoms | NA | NA | NA | NA | Steroids | Antibiotics | 1 |
| P60 | F | 63 | Encephalopathy | Blurred vision | NA | NA | NA | NA | None | None | 1 |
| P61 | M | 56 | Demyelinating encephalopathy | Motor disorder, speech disturbance | 4 | 3.5 | 0.29 | 119.8 | None | None | 1 |
| P62 | M | 62 | Demyelinating encephalopathy | Paresthesia | 0 | 4.29 | 0.48 | 128.5 | None | None | 3 |
| P63 | M | 52 | Metabolic encephalopathy | Disturbance of consciousness, cognitive dysfunction, motor disorder, speech disturbance, involuntary movement | 0 | 4.33 | 0.44 | 136.8 | Steroids | None | 2 |
| P64 | M | 65 | Demyelinating encephalopathy | Cognitive dysfunction, motor disorder, speech disturbance | NA | NA | NA | NA | None | Hyperbaric oxygen therapy | 1 |
| P65 | M | 52 | Encephalopathy | Fever, disturbance of consciousness, involuntary movement | 5 | 4.51 | 0.3 | 124 | None | Antibiotics, hyperbaric oxygen therapy | 3 |
| P66 | M | 23 | Encephalopathy | Headache, nausea, vomiting, motor disorder | 0 | 3.33 | 0.3 | 118.6 | None | Antibiotics | 1 |

**Abbreviations**: CRMPs, collapsin response mediator proteins; CSF, cerebrospinal fluid; EB, Epstein-Barr; F, female; GFAP, glial fibrillary acidic protein; Glu, glucose; HSV, herpes simplex virus; IVIG, intravenous immunoglobulin; M, male; MOG, myelin oligodendrocyte glycoprotein; mRS, modified Rankin Scale; NA, not available; NMDAR, N-methyl-D-aspartate receptor; Pro, protein; WBC, white blood cell.
