# Supplementary material for: Autoantibodies Against Collapsin Response Mediator Proteins Associated With Encephalopathy/Myelopathy: A Single‐Center Retrospective Study
Source: CNS Neurosci Ther. 2025 Jun 29;31(6):e70423. doi: 10.1111/cns.70423 (PMC12206659; doi:10.1111/cns.70423)
Supplement: Supplementary file 2 — Table S2. [file CNS-31-e70423-s002.docx]

**Supplementary Table 2 The imaging data of 22 patients with positive anti-CRMPs antibodies.**

| Patients | MRI or CT findings |
| --- | --- |
| P1 | MRI: suspicious abnormal singals in conus medullaris |
| P2 | MRI: mild white matter degeneration |
| P3 | CT: absent right skull and strip high-density signals considering the changes after intracranial surgery; low density signals of the right frontotempral lobe |
| P4 | MRI: abnormal signals of left temporal lobe and right parietal temporal junction area |
| P5 | MRI: increased and thickened blood vessels on brain surface |
| P6 | MRI: space occupying lesions in bilateral cerebellopontine angle cistern and cerebellar medullary cistern; diffuse leptomeningeal enhancement, considering meningitis |
| P7 | MRI: mild leukoaraiosis |
| P8 | MRI: Thickening of bilateral cerebellar tentorium, increased vessels on the surface of bilateral cerebellar hemispheres, and strip like high signal in the sulcus of the frontal, parietal, and occipital lobes, suggesting meningitis |
| P9 | MRI: multiple lesions of pons, right pontine, paraventricular area, left centrum semiovale, and genu of corpus callosum |
| P10 | MRI: cranial image was normal; cystic long T2 signal lesion of right adnexal area |
| P11 | MRI: abnormal signals of left frontal lobe, suggesting meningioencephalitis |
| P12 | MRI: mild white matter degeneration and brain atrophy |
| P13 | MRI: brain atrophy |
| P14 | MRI: symmetrical abnormal signals in bilateral insular subcortical white matter, hippocampus and parahippocampal gyrus |
| P15 | MRI: abnormal signals in bilateral cerebrum, cerebellum, brainstem; segmental lesions of thoracic spinal cord, considered as demyelinating lesions |
| P16 | MRI: multiple lesions of white matters, pons, considered as demyelinating lesions |
| P17 | MRI: segmental lesions of C5-C6 spinal cord, considered as demyelinating lesions |
| P18 | MRI: abnormal signals of left cingulate gyrus |
| P19 | MRI: on T2WI, high signals in bilateral radiative coronal area, considered as white matter degeneration; mild brain atrophy |
| P20 | MRI: white matter degeneration |
| P21 | MRI: spinal cord strip like abnormal signal , suspected demyelinating lesion |
| P22 | MRI: multiple patchy abnormal signals in bilateral frontal lobes, bilateral semiovral centers, bilateral periventricles and bilateral basal ganglia, suspected inflammation or demyelination |

**Abbreviations**: CRMPs, collapsin response mediator proteins; CT, computed tomography; MRI, magnetic resonance imaging.
